# A new filter QP-free method for the nonlinear inequality constrained optimization problem

**DOI:** 10.1186/s13660-018-1851-3

**Published:** 2018-10-11

**Authors:** Youlin Shang, Zheng-Fen Jin, Dingguo Pu

**Affiliations:** 1 School of Mathematics and Statistics, Henan University of Science and Technology, Luoyang, China; 2 Department of Mathematics, Tongji University, Shanghai, China

**Keywords:** 90C20, 90C30, 90C33, Nonlinear constrained optimization, Filter method, QP-free method, Nonmonotone line search

## Abstract

In this paper, a filter QP-free infeasible method with nonmonotone line search is proposed for minimizing a smooth optimization problem with smooth inequality constraints. This proposed method is based on the solution of nonsmooth equations, which are obtained by the Lagrangian multiplier method and the function of the nonlinear complementarity problem for the Karush–Kuhn–Tucker optimality conditions. Especially, each iteration of this method can be viewed as a perturbation of a Newton or quasi-Newton iteration on both the primal and dual variables for the solution of the Karush–Kuhn–Tucker optimality conditions. What is more, it is considered to use the function of the nonlinear complementarity problem in the filter, which makes the proposed algorithm avoid the incompatibility. Then the global convergence of the proposed method is given. And under some mild conditions, the superlinear convergence rate can be obtained. Finally, some preliminary numerical results are shown to illustrate that the proposed filter QP-free infeasible method is quite promising.

## Introduction

In this paper, we mainly consider solving the nonlinear optimization problem (NLP) with the inequality constraints, where the objective function and the constrained functions are Lipschitz continuously differentiable functions. We give the Lagrangian function associated with this problem, then the *Karush–Kuhn–Tucker* (KKT) optimality conditions for our solved problem can be obtained.

It is well known that the KKT optimality conditions is a mixed nonlinear complementarity problem (NCP). And this NCP has attracted much attention due to its various applications [1–3] such as the economic equilibrium problem, the restructuring problems of electricity and gas markets, and so on. Of course, there are many efficient methods for solving the NCP, which can be seen in [4–7]. One popular way to solve the NCP is to construct a Newton method for solving the related nonlinear equations, which is a reformulation of the KKT optimality condition. Another way is to use the filter method to directly solve the NLP with the inequality constraints. Recently Pu, Li, and Xue [8] proposed a new quadratic programming (QP)-free infeasible method for minimizing a smooth function subject to some inequality constraints. This method is based on the solution of nonsmooth equations which are obtained by the multiplier and the Fischer–Burmeister NCP function for the KKT conditions. They proved that the method had a superlinear convergence rate under some mild conditions.

Fletcher and Leyffer [9] proposed a filter method for solving the NLP problem, which was an alternative to the traditional merit functions approach. Provided that there is a sufficient decrease in the objective function or the constraints violation function, it was shown that the trial points generated from solving a sequence of trust region QP subproblems are accepted. In addition, the computational results reported in [9, 10] are also very encouraging. For more related methods, one can refer to [11–16].

Stimulated by the progress in these two aspects, in this paper, we propose a nonmonotone filter QP-free infeasible method for minimizing a smooth function subject to smooth inequality constraints. This proposed iterative method is based on the solution of nonsmooth equations, which are obtained by the multiplier and some NCP functions for the KKT first order optimality conditions. And each iteration of this method can be viewed as a perturbation of a Newton or quasi-Newton iteration on both the primal and dual variables for the solution of the KKT optimality conditions. Specifically, we use the filter on the linear search with a nonmonotone acceptance mechanism [17, 18]. Moreover, we also consider to use the NCP function in the filter. Thus our algorithm can avoid the incompatibility, which may appear in the filter SQP algorithm. We also give the global convergence and the superlinear convergence rate of the proposed method under some mild conditions. Finally, we take some numerical tests to illustrate the effectiveness of the proposed filter QP-free infeasible method.

The rest of this paper is organized as follows. In Sect. 2, we give some preliminaries and the formulation of the solved problem. Then we propose an infeasible filter QP-free method. In Sect. 3, we show that the proposed method is well defined and establish its global convergence and superlinear convergence rate under some mild conditions. Some numerical tests are given in Sect. 4. Finally, we give some brief conclusions in Sect. 5.

## Preliminaries and algorithm

In this section, we firstly introduce the formulation of the solved problem. Then we give some preliminaries for structuring a new filter QP-free method. Finally, we present the structure of our proposed method in detail.

In this paper, we mainly consider solving the nonlinear optimization problem (NLP) with the inequality constraints, which can be formulated as

1 $$ \min f(x), \quad \text{s.t.}\quad x\in D=\bigl\{ x\in R^{n}| G(x)\le0\bigr\} , $$

where $f:R^{n}\to R$ and $G=(g_{1},g_{2}, \ldots, g_{m})^{T}:R^{n}\to R^{m}$ are Lipschitz continuously differentiable functions.

The Lagrangian function associated with problem (1) is

$$L( x, \mu)=f(x)+\mu^{T}G(x), $$

where $\mu= (\mu_{1},\mu_{2},\ldots,\mu_{m})^{T}\in R^{m}$ is the multiplier vector. For simplicity, we use $(x,\mu)$ to denote the column vector $(x^{T},\mu^{T})^{T}$.

Then we can obtain the KKT point $(\bar{x}, \bar{\mu})\in R^{n}\times R^{m}$ for problem (1), which satisfies the necessary optimality conditions :

2 $$ \nabla_{x}L(\bar{x}, \bar{\mu})=0, \qquad G(\bar{x})\le0, \qquad \bar{\mu}\ge0,\qquad \bar{\mu}_{i}g_{i}(\bar{x})=0, $$

where $1\le i\le m$. We also say that $\bar{x}\in D$ is a KKT point of problem (1) if there exists $\bar{\mu}\in R^{m}$ such that $(\bar{x}, \bar{\mu})$ satisfies (2). It is well known that the KKT optimality condition is a mixed NCP. And the reformulation of (2) can be viewed as the following nonlinear equation:

$$ \Phi(x, \mu)=0. $$

### Preliminaries

In this subsection, we give the definition of Fischer–Burmeister NCP function and some related Jacobian functions in different cases. Both theoretical results and computational experience have indicated that the nonsmooth methods based on the Fischer–Burmeister NCP function are efficient. The Fischer–Burmeister function has a very simple structure, which is defined as

$$\psi(a, b)=\sqrt{a^{2}+b^{2}}-a-b. $$

It is clear that this function *ψ* is continuously differentiable everywhere except at the origin, but it is strongly semismooth at the origin, *i.e.*, if $a\neq0$ or $b\neq0$, then *ψ* is continuously differentiable at $(a, b)\in R^{2}$, and

$$\nabla\psi(a,b)= \biggl(\frac{a}{\sqrt{a^{2}+b^{2}}}-1, \frac{b}{\sqrt {a^{2}+b^{2}}}-1 \biggr); $$

if $a=0$ and $b=0$, then the generalized Jacobian of *ψ* at $(0,0)$ is (see [14])

$$\partial\psi(0,0)=\bigl\{ (\xi-1,\eta-1)| \xi^{2}+\eta^{2}=1 \bigr\} . $$

Let $\phi_{i}(x, \mu)=\psi(-g_{i}(x), \mu_{i})$, $1\le i\le m$. Given the above formulation of problem (1), we can denote $\Phi (x, \mu)=((\nabla_{x}L(x, \mu))^{T}, (\Phi_{1}(x, \mu))^{T})^{T}$, where $\Phi_{1}(x, \mu)=( \phi_{1}(x, \mu), \ldots , [4] \phi_{m}(x, \mu) )^{T}$.

Clearly, the KKT optimality conditions (2) can be equivalently reformulated as the nonsmooth equations $\Phi(x, \mu)=0$.

If $(g_{i}(x), \mu_{i})\neq(0,0)$, then $\phi_{i}$ is continuously differentiable at $(x, \mu)\in R^{n+m}$. In this case, we have

$$\nabla_{x}\phi_{i}= \biggl(\frac{-g_{i}(x)}{\sqrt{(g_{i}(x))^{2}+\mu_{i}^{2}}}+1 \biggr) \nabla g_{i}(x); \qquad \nabla_{\mu}\phi_{i}= \biggl(\frac{\mu_{i}}{\sqrt{(g_{i}(x))^{2}+\mu_{i}^{2}}}-1 \biggr)e_{i}, $$

where $e_{i}=(0,\ldots, 0,1,0,\ldots, 0)^{T}\in R^{m}$ is the *i*th column of the unit matrix, its *i*th element is 1, and other elements are 0.

If $g_{i}(x)=0$ and $\mu_{i}=0$, $1\le i\le m$, then $\phi_{i}(x, \mu)$ is strongly semismooth and directionally differentiable at $(x, \mu)$. We have

$$\partial_{x}\phi_{i}(x, \mu)=\bigl\{ (\xi+1)\nabla g_{i}(x)|-1\le\xi\le1\bigr\} $$

and

$$\partial_{\mu_{i}}\phi_{i}(x, \mu)=\bigl\{ (\xi-1)|-1\le\xi\le1 \bigr\} . $$

We may reformulate the KKT at point $(\bar{x}, \bar{\mu})$ conditions as a system of equations:

$$\Phi(\bar{x}, \bar{\mu})=\bigl(\nabla_{x}L(\bar{x}, \bar{\mu}), \Phi_{1}(\bar{x},\bar{\mu})\bigr)= 0, $$

where $\mu=(\mu_{1}, \mu_{2},\ldots,\mu_{m})^{T}\in R^{m}$ is the multiplier vector, $\phi_{j}(x,\mu_{j})=\psi(-g_{j}(x), \mu_{j})$, $\Phi_{1}(\bar{x},\bar{\mu})=(\phi_{1}(\bar{x},\bar{\mu}_{1}), \phi_{2}(\bar{x},\bar{\mu}_{2}),\ldots ,\phi_{m}(\bar{x},\bar{\mu}_{m}))^{T}$. To replace the violation constrained function $p(G(x))$ in the filter *F* of Fletcher and Leyffer method [9], we use the violation constrained function $p(G(x),\mu)=\|\Phi_{1}(x,\mu)\|$.

### Algorithm

In this subsection, we give the process and the framework of the filter QP-free method for solving problem (1). We firstly give some closed forms for preparing the method.

If $(g(x^{k}), \mu^{k})\neq(0,0)$, let $\xi_{j}^{k}=\xi_{j}(x^{k}, \mu^{k})=\frac{-g_{j}^{k}}{\sqrt{(g_{j}^{k})^{2}+(\mu^{k}_{j})^{2}}}+1$; $\eta_{j}^{k}=\eta_{j}(x^{k}, \mu^{k})=\frac{\mu^{k}_{j}}{\sqrt{(g_{j}^{k})^{2}+(\mu^{k}_{j})^{2}}}-1$; otherwise we denote $\xi_{j}^{k}=\xi_{j}(x^{k}, \mu^{k})=1+\sqrt{2}/2$; $\eta_{j}^{k}=\eta_{j}(x^{k}, \mu^{k})=-1+\sqrt{2}/2$. Then let

3 $$V^{k}=\left(\begin{array}{cc} V_{11}^{k} & V_{12}^{k}\\ V_{21}^{k} & V_{22}^{k} \end{array}\right)=\left(\begin{array}{cc} H^{k} & \nabla G^{k}\\ \mathrm{diag}(\xi^{k}){\left(\nabla G^{k}\right)}^{T} & \mathrm{diag}(\eta^{k}-c^{k}) \end{array}\right),$$

where $H^{k}$ is a positive matrix, which may be modified by BFGS update. The $\operatorname {diag}(\xi^{k})$ or $\operatorname{diag}(\eta^{k}-c^{k})$ denotes the diagonal matrix whose *j*th diagonal element is $\xi_{j}^{k}$ or $\eta_{j}^{k}-c_{j}^{k}$ respectively, and

$$c_{j}^{k} = c \min\bigl\{ 1, \bigl\Vert \Phi^{k} \bigr\Vert ^{\nu}\bigr\} , $$

where $c>0$ and $\nu>1$ are given parameters.

Secondly, we give the nonmonotone sequence for structuring our method. We may assume that the elements $\Phi^{k}$ and $F^{k}$ are sorted in the decreasing order, that is, $\hat{F}^{k1}\ge\hat{F}^{k2}\ge\hat{F}^{k3}\ge\cdots\ge\hat{F}^{kl}$, $\hat{\Phi}^{k1}\ge\hat{\Phi}^{k2}\ge\hat{\Phi}^{k3}\ge\cdots\ge\hat{\Phi}^{kl}$. Let

4 $$ \bar{\Phi}^{k}= = \textstyle\begin{cases} \{\Phi^{k}, \hat{\Phi}^{k2} , \hat{\Phi}^{k3},\ldots, \hat{\Phi}^{kl}\},&\mbox{if } \Vert \Phi^{k} \Vert < \hat{\Phi}^{k1} \mbox{ and } \Vert \Phi^{k} \Vert >0,\\ \{\hat{\Phi}^{k1}, \hat{\Phi}^{k2} , \hat{\Phi}^{k3},\ldots, \hat{\Phi}^{kl}\}, & \mbox{if }\Phi^{k}\ge\hat{\Phi}^{k1} \mbox{ or } \Phi^{k}=0; \end{cases} $$

and

5 $$ \bar{F}^{k}= = \textstyle\begin{cases} \{F^{k}, \hat{F}^{k2} , \hat{F}^{k3},\ldots, \hat{F}^{kl}\}, & \mbox{if }F^{k}< \hat{F}^{k1},\\ \{\hat{F}^{k1}, \hat{F}^{k2} , \hat{F}^{k3},\ldots, \hat{F}^{kl}\}, &\mbox{if } F^{k}\ge\hat{F}^{k1}. \end{cases} $$

We denote the maximal elements in $\bar{\Phi}^{k}$, $\bar{F}^{k}$ by $p^{k}_{\max}$, $\bar{F}^{k}_{\max}$, respectively.

Based on the above given information, we now give the framework of the nonmonotone filter QP-free infeasible method (NFQPIM) for minimizing a smooth function subject to smooth inequality constraints as follows in Algorithm 1.

**Algorithm 1:**
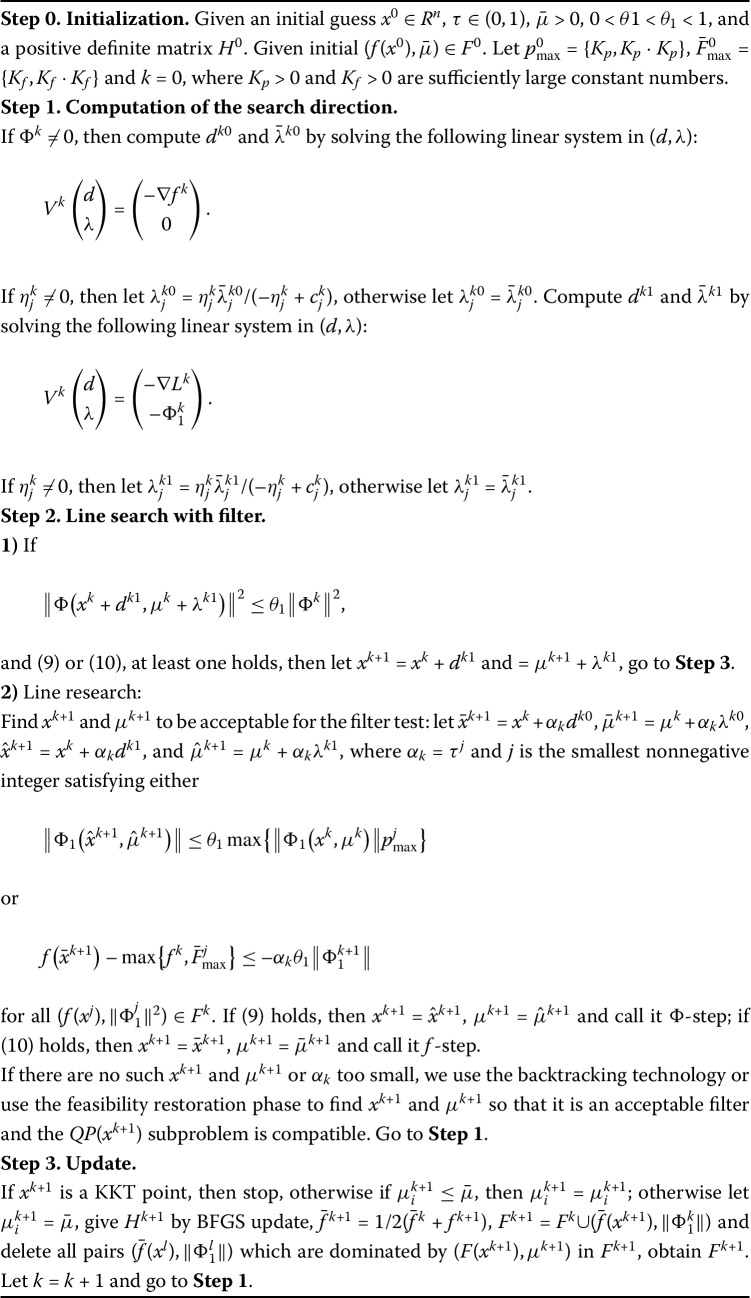
NFQPIM

#### Remark 1

Let $\Phi(x, \mu)=((\nabla_{x}L, H(x)),(\Phi_{1}(x, \mu))^{T})^{T}$, the above proposed NFQPIM can also be used to solve the following constrained NLP:

$$\begin{aligned} &\min\mbox{ }f(x) \\ &\text{s.t.}\quad G(x)\le0, \qquad H(x)=0, \quad x\in R^{n}, \end{aligned}$$

where $f:R^{n}\to R$ and $G(x)=(g_{1}(x),g_{2}(x), \ldots, g_{m}(x))^{T}:R^{n}\to R^{m}$ and $H(x)=(h_{1}(x),h_{2}(x), \ldots, h_{p}(x))^{T}:R^{p}\to R^{m}$ are Lipschitz continuously differentiable functions.

### Implementation

In this subsection, we give the implementation of the proposed NFQPIM. Firstly, we suppose that the following assumptions A1–A3 hold.

A1.: The level set $\{x|f(x)\le f(x^{0})\}$ is bounded, and for sufficiently large *k*, $\|\mu^{k}+\lambda^{k0}+\lambda^{k1}\|< \bar{\mu}$.
A2.: *f* and $g_{i}$ are Lipschitz continuously differentiable, and for all *y*, $z\in R^{n+m}$,
  $$\bigl\Vert \nabla L(y)-\nabla L(z) \bigr\Vert \le m_{0} \Vert y-z \Vert , \qquad \bigl\Vert \Phi(y)-\Phi(z) \bigr\Vert \le m_{0} \Vert y-z \Vert , $$
  where $m_{0}>0$ is the Lipschitz constant.
A3.: $H^{k}$ is positive definite and there exist positive numbers $m_{1}$ and $m_{2}$ such that $m_{1}\|d\|^{2}\le d^{T}H^{k}d\le m_{2}\|d\|^{2}$ for all $d\in R^{n}$ and all *k*.

#### Lemma 1

*If* $\Phi^{k} \neq0$, *then* $V^{k} $ *is nonsingular*.

#### Proof

Assume $\Phi^{k} \neq0$. If $V^{k}(u, v)=0$ for some $(u,v)\in R^{n+m}$, where $u=(u_{1},\ldots, u_{n})^{T}$, $v=(v_{1},\ldots, v_{m})^{T}$, then

6 $$ H^{k}u+\nabla G^{k}v=0 $$

and

7 $$ \operatorname{diag}\bigl(\xi^{k}\bigr) \bigl(\nabla G^{k} \bigr)^{T}u+\operatorname{diag}\bigl(\eta ^{k}-c^{k} \bigr)v=0. $$

From the definitions of $\xi_{j}^{k}$ and $\eta_{j}^{k}$, we know that $\xi_{j}^{k}\ge0$ and $\eta_{j}^{k} -c^{k}\neq0$ for all *j*. So $\operatorname{diag}(\eta^{k}-c_{j}^{k})$ is nonsingular. We have

8 $$ v=-\bigl(\operatorname{diag}\bigl(\eta^{k}-c^{k}\bigr) \bigr)^{-1}\operatorname{diag}\bigl(\xi ^{k}\bigr) \bigl( \nabla G^{k}\bigr)^{T}u . $$

Taking (13) into (11), we have

$$u^{T}\bigl(H^{k}u+\nabla G^{k}v\bigr) =u^{T}H^{k}u- u^{T}\nabla G^{k} \operatorname{diag}\bigl(\xi ^{k}\bigr) \bigl(\operatorname{diag}\bigl( \eta^{k}-c^{k}\bigr)\bigr)^{-1}\bigl(\nabla G^{k}\bigr)^{T}u=0. $$

The fact that $-\nabla G^{k}\operatorname{diag}(\xi^{k})(\operatorname {diag}(\eta^{k}-c^{k}))^{-1}(\nabla G^{k})^{T}$ is positive semidefinite implies $u=0$, and then $v=0$ by (13). $V^{k} $ is nonsingular. This lemma holds. □

#### Lemma 2

$d^{k0}=0$ *if and only if* $\nabla f^{k}=0$, *and* $d^{k0}=0$ *implies* $\bar{\lambda}^{k0}=0$ *and* $\lambda^{k0}=0$.

*If* $(x^{*}, \mu^{*})$ *is an accumulation point of* $\{( x^{k},\mu^{k})\}$, *then* $d^{*0}=0$, *and* $(d^{*0},\lambda^{*0})^{T}$ *is the solution of the following equations*:

9 $$V^{*}\left(\begin{array}{c} d\\ \lambda \end{array}\right)=\left(\begin{array}{c} -\nabla f^{*}\\ 0 \end{array}\right)$$

*and* $\nabla L(x^{*},\mu^{*})=0$.

It is clear that the following lemma holds, with reference to [8].

#### Lemma 3

*If* $d^{k0}\neq0$, *then*

$$\bigl(d^{k0}\bigr)^{T}H^{k}d^{k0}\le- \bigl(d^{k0}\bigr)^{T}\nabla f^{k}. $$

#### Proof

(14) implies

10 $$ H^{k}d^{k0}+\nabla G^{k}\lambda^{k0}=- \nabla f^{k}, $$

and

11 $$ \operatorname{diag}\bigl(\xi^{k}\bigr) \bigl(\nabla G^{k} \bigr)^{T}d^{k0}+\operatorname{diag}\bigl(\eta^{k}- c^{k}\bigr)\hat{\lambda}^{k0}=0. $$

We have

12 $$ \hat{\lambda}^{k0}=-\bigl(\operatorname{diag}\bigl(\eta^{k}- c^{k}\bigr)\bigr)^{-1}\operatorname{diag}\bigl( \xi^{k}\bigr) \bigl(\nabla G^{k}\bigr)^{T}d^{k0} . $$

Taking (17) into (15), we have

13 $$\begin{aligned} &\bigl(d^{k0}\bigr)^{T}\bigl(H^{k}d^{k0}+ \nabla G^{k}\lambda^{k0}\bigr) \\ &\quad =\bigl(d^{k0}\bigr)^{T}H^{k}d^{k0}- \bigl(d^{k0}\bigr)^{T}\nabla G^{k} \operatorname{diag}\bigl(\xi ^{k}\bigr) \bigl(\operatorname{diag}\bigl( \eta^{k}- c^{k}\bigr)\bigr)^{-1}\bigl(\nabla G^{k}\bigr)^{T}d^{k0} \\ &\quad =-\bigl(d^{k0}\bigr)^{T}\nabla f^{k}. \end{aligned}$$

$(d^{k0})^{T}\nabla G(x^{k})\operatorname{diag}(\xi^{k})(\operatorname {diag}(\eta^{k}-c^{k}))^{-1}(\nabla G^{k})^{T}d^{k0}\le0$ implies

14 $$ \bigl(d^{k0}\bigr)^{T}H^{k}d^{k0}\le- \bigl(d^{k0}\bigr)^{T}\nabla f^{k}. $$

The lemma holds. □

#### Lemma 4

*There exists* $m_{3}>0$ *such that*, *for any* $0< t\le1$,

$$\bigl\Vert \Phi_{1}\bigl(x^{k}+td^{k0}, \mu^{k}+t\lambda^{k0}\bigr) \bigr\Vert ^{2}- \Vert \Phi_{1} \Vert ^{2} \le m_{3} t^{2}. $$

#### Proof

If $\Phi_{1}^{k}=0$, then there exists $m_{4}>0$ such that, for any $0< t\le1$,

$$\begin{aligned} \bigl\Vert \Phi_{1}\bigl(x^{k}+td^{k0}, \mu^{k}+t\lambda^{k0}\bigr) \bigr\Vert ^{2} &= \bigl\Vert \Phi_{1}\bigl(x^{k}+td^{k0}, \mu^{k}+t\lambda^{k0}\bigr)-\Phi_{1}^{k} \bigr\Vert ^{2} \le t^{2}m_{2}^{2} \bigl\Vert \bigl(d^{k0},\lambda^{k0}\bigr) \bigr\Vert ^{2}. \end{aligned} $$

The lemma holds for $\Phi_{1}^{k}=0$. □

We define that if $(g_{i}^{k},\mu_{i}^{k})\neq(0,0)$, then $(\bar{\xi}_{i}^{k0},\bar{\eta}_{i}^{k0})=(\xi_{i}^{k},\eta_{i}^{k})$, otherwise $\bar{\xi}_{i}^{k0}(\nabla g_{i}^{k})^{T}d^{k0}+\bar{\eta}_{i}^{k0} \lambda_{i}^{k0}= \phi_{i}'((x^{k},\mu^{k}), (d^{k0}, \lambda^{k0}))$, where $\phi_{i}'((x^{k},\mu^{k}), (d^{k0}, \lambda^{k0}))$ is the direction derivative of $\phi_{i}(x,\mu )$ at $(x^{k},\mu^{k})$ in the direction $(d^{k0}, \lambda^{k0})$.

Let $\operatorname{diag}(\bar{\xi}^{k0})$ or $\operatorname{diag}(\bar{\eta}^{k0})$ denote the diagonal matrix whose *j*th diagonal element is $\bar{\xi}_{j}^{k0}$ or $\bar{\eta}_{j}^{k0}$, respectively. Then $\phi_{i}(0,0)=0$ implies

$$\bigl(\Phi_{1}^{k}\bigr)^{T}\bigl( \operatorname{diag}\bigl(\bar{\xi}^{k0}\bigr) \bigl(\nabla G^{k} \bigr)^{T}, \operatorname{diag}\bigl(\bar{\eta}^{k0}\bigr) \bigr)=\bigl(\Phi_{1}^{k}\bigr)^{T}\bigl( \operatorname{diag}\bigl(\xi^{k}\bigr) \bigl(\nabla G^{k} \bigr)^{T}, \operatorname{diag}\bigl(\eta^{k}\bigr)\bigr). $$

Then

15 $$\begin{aligned} & \bigl\Vert \Phi_{1}^{k}+t\bigl(\operatorname{diag} \bigl(\bar{\xi}^{k0}\bigr) \bigl(\nabla G^{k} \bigr)^{T}d^{k0}+ \operatorname{diag}\bigl(\bar{\eta}^{k0}\bigr)\lambda^{k0}\bigr) \bigr\Vert ^{2} \\ &\quad = \bigl\Vert \Phi_{1}^{k} \bigr\Vert ^{2}+t^{2}\| \operatorname{diag}\bigl(\bar{\xi}^{k0} \bigr) \bigl(\nabla G^{k}\bigr)^{T}d^{k0}+ \operatorname{diag}\bigl(\bar{\eta}^{k0}\bigr)\lambda^{k0}) \|^{2}. \end{aligned}$$

It is clear that

$$\bigl\Vert \Phi_{1}\bigl(x^{k}+td^{k0}, \mu^{k}+t\lambda^{k0}\bigr) \bigr\Vert ^{2}= \bigl\Vert \Phi_{1}^{k} \bigr\Vert ^{2}+O \bigl(t^{2}\bigr). $$

This lemma holds.

#### Lemma 5

*If* $\Phi_{1}^{k}\neq0$, *then given any* $\varepsilon>0$ *there is* $\bar{t}>0$ *such that*, *for any* $0< t\le\bar{t}$,

$$\bigl\Vert \Phi_{1}^{k} \bigr\Vert ^{2}- \bigl\Vert \Phi_{1}\bigl(x^{k}+td^{k1}, \mu^{k}+t\lambda^{k1}\bigr) \bigr\Vert ^{2} \ge(2- \varepsilon)t \bigl\Vert \Phi_{1}^{k} \bigr\Vert ^{2}. $$

#### Proof

If $\Phi_{1}^{k}\neq0$, (7) implies

16 $$ \operatorname{diag}\bigl(\xi^{k}\bigr) \bigl(\nabla G^{k} \bigr)^{T}d^{k1}+\operatorname{diag}\bigl( \eta^{k}-c^{k}\bigr)\lambda^{k1}=- \Phi_{1}^{k}. $$

We define that if $(g_{i}^{k},\mu_{i}^{k})\neq(0,0)$ then $(\bar{\xi}_{i}^{k1},\bar{\eta}_{i}^{k1})=(\xi_{i}^{k},\eta_{i}^{k})$, otherwise $(\bar{\xi}_{i}^{k1}\nabla g_{i}^{k}, \bar{\eta}_{i}^{k1})(d^{k1}, \lambda^{k1})= \phi_{i}'((x^{k},\mu^{k}), (d^{k1}, \lambda^{k1}))$, where $\phi_{i}'((x^{k},\mu^{k}), (d^{k1}, \lambda^{k1}))$ is the direction derivative of $\phi_{i}(x,\mu )$ at $(x^{k},\mu^{k})$ in the direction $(d^{k1}, \lambda^{k1})$. Let $\operatorname{diag}(\bar{\xi}^{k1})$ or $\operatorname{diag}(\bar{\eta}^{k1})$ denote the diagonal matrix whose *i*th diagonal element is $\bar{\xi}_{i}^{k1}$ or $\bar{\eta}_{i}^{k1}$, respectively. Clearly, for all *i*,

17 $$ \phi_{i}\bigl(x^{k}+td^{k1},\mu^{k}+t \lambda^{k1}\bigr) -\phi_{i}^{k}\leq t\bigl(\bar{\xi}_{i}^{k1}\bigl(\nabla g_{i}^{k} \bigr)^{T}d^{k1}+\bigl(\bar{\eta}_{i}^{k1} \bigr)\bigr). $$

Since $c^{k}_{i}\neq0$, it follows by the definitions of $c^{k}_{i}$ and $\eta_{i}^{k}$ that $\eta_{i}^{k}=0$, $g_{i}^{k}=0$, $\mu_{i}^{k}\ge0$, and $\phi_{i}^{k}=0$. We have

18 $$\begin{aligned} & \bigl\Vert \Phi_{1}^{k}+t\bigl(\operatorname{diag} \bigl(\bar{\xi}^{k1}\bigr) \bigl(\nabla G^{k} \bigr)^{T}d^{k1}+ \operatorname{diag}\bigl(\bar{\eta}^{k1} \bigr)\lambda^{k1}\bigr) \bigr\Vert ^{2} \\ &\quad = (1-2t) \bigl\Vert \Phi_{1}^{k} \bigr\Vert ^{2}+ t \bigl\Vert \operatorname{diag}\bigl(\bar{\xi}^{k1} \bigr) \bigl(\nabla G^{k}\bigr)^{T}d^{k1} + \operatorname{diag}\bigl(\bar{\eta}^{k1}\bigr)\lambda^{k1} \bigr\Vert ^{2}. \end{aligned}$$

It follows from (22) and (23) that, given any $\varepsilon>0$, there is $\bar{t}>0$ such that, for any $0< t\le\bar{t}$,

$$\bigl\Vert \Phi_{1}^{k} \bigr\Vert ^{2}- \bigl\Vert \Phi_{1}\bigl(x^{k}+t^{2}d^{k1}, \mu^{k}+t \lambda^{k1}\bigr) \bigr\Vert ^{2}\ge(2- \varepsilon)t \bigl\Vert \Phi_{1}^{k} \bigr\Vert ^{2}. $$

Hence this lemma holds. □

#### Lemma 6

$d^{k0}=0$ *if and only if* $\nabla f^{k}=0$, *and* $d^{k0}=0$ *implies* $\bar{\lambda}^{k0}=0$ *and* $\lambda^{k0}=0$.

#### Proof

If $\nabla f^{k}=0$, then $(x^{k},\bar{\lambda}^{k0})=\hat{V}^{k}(0,0)=(0,0)$. If $d^{k0}=0$, then (14) implies

19 $$ \operatorname{diag}\bigl(\xi^{k}\bigr) \bigl(\nabla G^{k} \bigr)^{T}d^{k0}+\operatorname{diag}\bigl( \eta^{k}-c^{k}\bigr) \bar{\lambda}^{k0}= \operatorname{diag}\bigl(\eta^{k}-c^{k}\bigr)\bar{\lambda}^{k0}=0. $$

Clearly, $\bar{\lambda}^{k0}=0$, $\lambda^{k0}=0$, and $(\nabla f^{k},0)=(V^{k})^{-1}(0,0)=(0,0)$. □

From Lemmas 3–6, we know that, if $\Phi _{1}^{k}\neq0$, then $(d^{k},\lambda^{k})$ is the decreasing direction of $\|\Phi^{k}\|$; if $d^{k0}\neq0$, then $d^{k}$ is the decreasing direction of $f^{k}$. If $\Phi_{1}^{k}=0$ and $d^{k0}=0$, then $(x^{k},\mu^{k})$ is a KKT point. We consider four cases for linear searches.

*Case 1*. $k-1$ iteration has a Φ-step and $\Phi_{1}^{k}=0$. In this case, $p^{k}_{\max}=p^{k-1}_{\max}$ and $\min\{p^{k} j_{\max}|j\in F^{k}>0\}$. Clearly, we can find $\alpha _{k}$ such that $\hat{x}^{k+1}$ satisfies (9).

*Case 2*. $k-1$ iteration has a Φ-step and $\Phi_{1}^{k}\neq0$. In this case, it follows from Lemma 5 that, given any $\varepsilon>0$, there is $\bar{t}>0$ such that, for any $0< t\le\bar{t}$,

$$\bigl\Vert \Phi_{1}^{k} \bigr\Vert ^{2}- \bigl\Vert \Phi_{1}\bigl(x^{k}+td^{k1}, \mu^{k}+t\lambda^{k1}\bigr) \bigr\Vert ^{2} \ge(2- \varepsilon)t \bigl\Vert \Phi_{1}^{k} \bigr\Vert ^{2}. $$

$p^{k}_{\max}>0$ is monotonically nonincreasing. So, we can find $\alpha_{k}$ such that $\hat{x}^{k+1}$ satisfies (9).

*Case 3*. $k-1$ iteration has an *f*-step and $d^{k0}\neq0$. In this case, it follows from Lemma 5 that, if $d^{k0}\neq0$, then

$$\bigl(d^{k0}\bigr)^{T}H^{k}d^{k0}\le- \bigl(d^{k0}\bigr)^{T}\nabla f^{k}, $$

where $f^{k}_{\max}$ is monotonically nonincreasing. We can find $\alpha_{k}$ such that $\bar{x}^{k+1}$ satisfies (10).

*Case 4*. The $(k-1)$ iteration has an *f*-step and $d^{k0}=0$. In this case, if $\Phi_{1}^{k}=0$, then $(x^{k}, \mu^{k} )$ is a KKT point, otherwise $x^{k}$ may be an infeasible stationary point.

If there are no such $x^{k+1}$ and $\mu^{k+1}$ or $\alpha_{k}$ too small, we use the backtracking technology or use the feasibility restoration phase to find $x^{k+1}$ and $\mu^{k+1}$ so that it is acceptable that the filter and the $QP(x^{k+1})$ subproblem are compatible.

## Convergence

In this section, we discuss the global and superlinear convergence rate of the proposed method. We give the following A4 and suppose that the assumptions A1–A4 hold in this section.

A4.: For all *k* and some $\alpha_{\min}>0$, $\alpha_{k}>\alpha_{\min}>0$.

It implies from (3) and (4) that $p^{k}_{\max}>0$ is monotonically nonincreasing and, if $\|\Phi_{1}(x^{k})\| \to0$, then $p^{k}_{\max}\to0$.

### Lemma 7

*Consider the sequence* $\{\|\Phi_{1}(x^{k})\|^{2}\}$ *and* $\{f^{k}\}$ *such that* $\{\|\Phi_{1}(x^{k})\|^{2}\ge0\}$ *and* $\{f^{k}\}$ *is monotonically decreasing and bounded below*. *Let a constant* *θ* *satisfy*, *for all* *k* *and* $l\in F ^{k}$, *that*

20 $$ \bigl\Vert \Phi_{1}\bigl(\hat{x}^{k+1}, \hat{\mu}^{k+1}\bigr) \bigr\Vert \le\theta_{1}\max\bigl\{ \bigl\Vert \Phi_{1}\bigl(x^{k},\mu^{k}\bigr) \bigr\Vert p^{j}_{\max}\bigr\} $$

*or*

21 $$ f\bigl(\bar{x}^{k+1}\bigr)-\max\bigl\{ f^{k},\bar{F}^{j}_{\max}\bigr\} \le-\alpha_{k} \theta_{1} \bigl\Vert \Phi _{1}^{k+1} \bigr\Vert , $$

*where* $\alpha_{k}\ge\alpha_{\min} >0$ *is the step length*, *θ* *is a given positive number*. *Then* $p^{k}_{\max}\to0$.

### Proof

Suppose that the theorem is not true, then $\Phi_{1}(x^{k})\not \to0$, and there exist $\varepsilon>0$ and infinitely many members of index set *K* such that $\|\Phi_{1}(x^{k+1},\mu^{k+1})\|\ge\varepsilon>0$, $p^{k}_{\max}\ge \varepsilon>0$, and $\|\Phi_{1}(x^{k+1},\mu^{k+1})\|\ge\theta\|\Phi_{1}(x^{k},\mu^{k})\|$ for any $k\in K$. We have

22 $$ f\bigl(x^{k}\bigr)-f\bigl(x^{k+1}\bigr)\ge\alpha_{k} \theta \bigl\Vert \Phi_{1}\bigl(x^{k+1},\mu ^{k+1} \bigr) \bigr\Vert > \alpha_{\min}\theta\varepsilon. $$

Because $\{f^{k}\}$ is monotonically decreasing, (27) implies $f(x^{k})\to-\infty$ as $k \to+\infty$, which is contravention of $\{f^{k}\}$ being bounded below. This lemma holds. □

### Lemma 8

*Consider an infinite sequence iterations on which* $\{f^{k},\|\Phi_{1}(x^{k})\|^{2}\}$ *entered into the filter*, *where* $\|\Phi _{1}(x^{k})\|>0$ *and* $\{f^{k}\}$ *is bounded below*. *It follows that* $\Phi_{1}(x^{k})\to0 $.

### Theorem 1

*If* $(x^{*}, \mu^{*})$ *is an accumulation point of* $\{( x^{k},\mu^{k})\}$, *then* $x^{*}$ *is a KKT point of problem* (1).

### Proof

It is obvious that Lemmas 8 and 2 imply that Theorem 1 holds. □

Next we consider the superlinear convergence of the method and firstly give the following assumptions we need.

A5.: The Mangasarian–Fromovitz (M-F) qualification condition is satisfied at $x^{*}$, *i.e.*, $\{\nabla g_{i}(x^{*})\}$ are linear independent for all $i\in I=\{i| g_{i}(x^{*})=0\}$, and there exists a direction such that $d^{T}\nabla g_{i}(x^{*})<0$, $i\in I=\{i| g_{i}(x^{*})=0\}$, where $i\in I=\{i| g_{i}(x^{*})=0\}$, where $x^{*}$ is an accumulation point of $\{x^{k}\}$ and a KKT point of problem (1).
A6.: The sequence of $\{H^{k}\}$ satisfies
  $$\frac{ \Vert (H^{k}-\nabla_{x}^{2}L(x^{k},\mu^{k} ))d^{k1} \Vert }{ \Vert d^{k1} \Vert }\to0. $$
A7.: The strict complementarity condition holds at each KKT point $(x^{*},\mu^{*} )$.

It follows that $\phi^{k}$ is differentiable at each KKT point $(x^{*},\mu^{*} )$.

Assumption A7 implies that Φ is continuously differentiable at each KKT point $(x^{*},\mu^{*} )$. As Lemma 1, we have that the following lemmas hold.

### Lemma 9

*Assume* A1–A7 *hold*, *then* $\{ \| (V^{k})^{-1}\| \}$ *and* $\{ \|(\hat{V}^{k})^{-1}\| \}$ *are bounded*. *Furthermore*, *if* $V^{*}$ *is an accumulation matrix of* $\{ V^{k}\}$, *then* $V^{*}$ *is nonsingular*.

### Proof

By Theorem 1, $\Phi^{*}=0$ and $c^{k}\to0$. Without loss of generality, we may assume that $( x^{k},\mu^{k})\to(x^{*}, \mu^{*})$, $H^{k}\to H^{*}$, $\operatorname{diag}(\xi^{k})\to\operatorname{diag}(\xi ^{*})$, and $\operatorname{diag} (\eta^{k})\to \operatorname{diag}(\eta^{*})$. By the definitions of $\xi_{i}^{k}$ and $\eta _{i}^{k}$, we know that $(\xi_{i}^{*})^{2}+(\eta_{i}^{*})^{2}\neq0$. $H^{k}\to H^{*}$ implies that $H^{*}$ is positive definite.

If $V^{*}(u, v)=0$, where $(u,v)\in R^{n+m}$ and $u=\{(u_{1},\ldots, u_{n})^{T}\}$, $v=\{(v_{1},\ldots, v_{m})^{T}\}$, then we have

23 $$ H^{*}u+\nabla G^{*}v=0 $$

and

24 $$ \operatorname{diag}\bigl(\xi^{*}\bigr) \bigl(\nabla G^{*}\bigr)^{T}u+ \operatorname{diag}\bigl(\eta ^{*}\bigr)v=0. $$

From (29) and the definitions of $\xi_{j}^{*}$ and $\eta_{j}^{*}$, we know that if $\xi_{j}^{*}=0$ then $\eta_{j}^{*}\neq0$. If $\xi_{j}^{*}\neq0$ then

25 $$ u^{T}\nabla g_{j}^{*}=-\frac{\eta_{j}^{*}}{\xi_{j}^{*}}v_{j} . $$

Putting (29) and (30) into (28), we have

26 $$\begin{aligned} &u^{T}\bigl( H^{*}u+\nabla G^{*}v\bigr) \\ &\quad =u^{T}H^{*}u+\sum_{j:\xi_{j}^{*}\neq0}- \frac{\eta_{j}^{*}}{\xi_{j}^{*}}v_{j}^{2}=0. \end{aligned}$$

$\eta_{j}^{*}/\xi_{j}^{*}\le0$ implies $u=0$, and if $\eta_{j}^{*}\neq0$ then $v_{j}=0$. Let $I= \{j| g_{j}^{*}=0\}$, because $g_{j}^{*}\neq0$ implies $\eta_{j}^{*}\neq0$ and $v_{j}=0$, we have

27 $$ \sum_{j\in I}\nabla g_{j}^{*}v_{j}=0, $$

and $v_{j}=0$ ($j\in I$) by A4, *i.e.*, $(u,v)=0$ and $V^{*}$ is nonsingular.

On the other hand, suppose to the contrary that there exists a subsequence $\{(x^{k(i)}, \lambda^{k(i)})\}$ such that $\|( V^{k(i)})^{-1}\|\to\infty$ as $k(i)\to\infty$ and $(x^{k(i)}, \lambda^{k(i)}) \to(x^{*}, \lambda^{*})$. We can choose $k(i)$ properly such that $V^{k(i)}\to V^{*}$ including $\xi^{k(i)}\to\xi^{*}$ and $\eta^{k(i)}\to\eta^{*}$. Clearly, $(\xi_{j}^{*})^{2}+ (\eta_{j}^{*})^{2}\ge3-2\sqrt{2}>0$ and $V^{*}\in\partial \Phi^{*}$. But $V^{*}$ is nonsingular by the above proof, which contradicts the assumption $\|( V^{k(i)})^{-1}\|\to\infty$. Hence, $\{ \|( V^{k(i)})^{-1}\| \}$ is bounded. $\Phi^{k}\to0$ implies $\lim_{k\to\infty}V^{k}=\lim_{k\to\infty}\hat{V}^{k}$, we can also obtain that $\{ \|( \hat{V}^{k})^{-1}\| \}$ is bounded. This lemma holds. □

Assumption A5 shows that $(x^{k}, \mu^{k})$ is a Newton direction with a high order perturbation. We obtain the following lemma.

### Lemma 10

*For sufficiently large* *k*, $x^{k+1}=x^{k}+d^{k1}$ *and* $\mu^{k+1}=\mu^{k}+\lambda^{k1}$.

Furthermore, Lemma 10 implies that the following theorem holds.

### Theorem 2

*Assume* A1–A7 *hold*. *Let Algorithm* 1 (*NFQPIM*) *be implemented to generate a sequence* $\{(x^{k},\mu^{k})\}$, $(x^{*}, \mu^{*})$ *be an accumulation point of* $\{(x^{k},\lambda^{k})\}$. *Then* $(x^{*}, \mu^{*})$ *is a KKT point of problem* (1), *and* $(x^{k},\mu^{k})$ *converges to* $(x^{*}, \mu^{*})$ *superlinearly*.

## Numerical tests

We use Algorithm 1 (NFQPIM) for the constrained optimization problems (see [19]): $H^{k}$ is updated by the BFGS method. The termination criterion is $\|\phi\|\le10^{-5}$. The parameters are chosen as follows: $c=0.1$, $\nu=2$, $\tau=0.7$, $\theta_{1}=0.8$, $\theta=0.6$, $\bar{\mu}=10\text{,}000$. In the “NIT/NG” entry of the table below, NIT is the number of iterations, NF represents the number of function evaluations, NG denotes the number of gradient evaluations. The numerical results can be seen in the Table 1. We test the proposed NFQPIM for solving almost 100 optimization problems. And the numerical results illustrate that the proposed method is efficient and promising.

**Table 1:** Numerical results on the NFQPIM for some constrained optimization problems

Problem	*n*	*m*	NIT	NG	NF
hs001	2	1	65	43	40
hs002	2	1	15	19	18
hs003	2	1	3	5	4
hs004	2	2	4	6	5
hs005	2	4	9	11	9
hs006	2	1	5	9	8
hs007	2	1	16	25	21
hs008	2	2	3	6	5
hs009	2	1	10	13	12
hs010	2	1	27	43	33
hs011	2	1	13	23	15
hs012	2	1	13	19	16
hs013	2	1	3	6	4
hs014	2	2	4	7	4
hs015	2	2	7	11	7
hs016	2	5	5	8	7
hs017	2	5	14	19	16
hs018	2	6	20	24	23
hs019	2	6	4	7	6
hs020	2	5	5	8	6
hs021	2	5	4	7	5
hs022	2	2	5	8	5
hs023	2	9	3	4	4
hs024	2	5	7	13	10
hs025	3	6	4	6	6
hs026	3	1	24	33	27
hs027	3	1	28	34	31
hs028	3	1	6	12	9
hs029	3	1	21	37	31
hs030	3	7	9	11	10
hs031	3	7	12	17	14
hs032	3	5	8	14	12
hs033	3	6	11	16	24
hs034	3	8	8	12	10
hs035	3	4	7	11	10
hs036	3	7	10	13	11
hs037	3	8	13	18	15
hs038	4	8	83	111	98
hs039	4	2	21	35	32
hs040	4	3	11	16	14
hs041	4	9	13	17	15
hs042	4	2	11	14	12
hs043	4	3	15	23	20
hs044	4	10	16	23	21
hs045	5	10	5	7	7
hs046	5	10	29	37	33
hs047	5	3	26	33	30
hs048	5	2	10	15	13
hs049	5	2	36	46	40
hs050	5	3	35	43	39
hs051	5	3	9	13	11
hs052	5	3	7	14	12
hs053	5	3	5	7	7
hs054	6	13	9	13	10
hs055	6	14	8	14	11
hs056	7	4	12	14	12
hs057	2	3	6	9	8
hs059	2	7	28	33	30
hs060	3	7	13	23	21
hs061	3	2	59	68	58
hs062	3	7	24	33	28
hs063	3	5	15	23	20
hs064	3	4	36	43	37
hs065	3	7	21	29	27
hs066	3	8	13	23	19
hs067	3	41	65	53	47
hs099	7	16	65	43	40
hs100	7	4	65	43	40
hs101	7	20	65	43	40
hs102	7	20	65	43	40
hs103	7	20	65	43	40
hs104	8	22	65	43	40
hs105	8	17	65	43	40
hs106	8	22	65	43	40
hs107	9	14	65	43	40
hs108	9	14	33	30	
hs110	10	20	25	43	40
hs111	10	23	61	73	68
hs112	10	13	51	73	67
hs113	10	8	55	65	61
hs114	10	31	56	73	68
hs116	13	41	123	143	140
hs117	15	20	511	63	60
hs118	15	59	67	81	77
hs119	16	40	68	83	78

## Conclusions

In this paper, we developed a nonmonotone filter QP-free infeasible method for minimizing a smooth optimization problem with inequality constraints. This proposed method is based on the solution of nonsmooth equations which are obtained by the multiplier and some NCP functions for the KKT first-order optimality conditions. At each iteration of the proposed method, it was a perturbation of a Newton or quasi-Newton iteration on both the primal and dual variables for the solution of the KKT optimality conditions. Moreover, we used the filter on linear searches with a nonmonotone acceptance mechanism. We also showed that the proposed method had a global convergence and a superlinear convergence rate. Finally, the numerical results illustrated that the proposed method was efficient. However, how to apply this method to the real optimal problem will be studied in the near future.
